# In-Depth, Label-Free Analysis of the Erythrocyte Cytoplasmic Proteome in Diamond Blackfan Anemia Identifies a Unique Inflammatory Signature

**DOI:** 10.1371/journal.pone.0140036

**Published:** 2015-10-16

**Authors:** Esther N. Pesciotta, Ho-Sun Lam, Andrew Kossenkov, Jingping Ge, Louise C. Showe, Philip J. Mason, Monica Bessler, David W. Speicher

**Affiliations:** 1 Division of Hematology, Department of Pediatrics, Children’s Hospital of Philadelphia, University of Pennsylvania, 3615 Civic Center Blvd Philadelphia, PA, 19104, United States of America; 2 Center for Systems and Computational Biology and Molecular and Cellular Oncogenesis Program, The Wistar Institute, 3601 Spruce St. Philadelphia, PA, 19104, United States of America; 3 Division of Hematology/Oncology, Department of Internal Medicine, University of Pennsylvania, 3620 Hamilton Walk Philadelphia, PA, 19104, United States of America; National Institute of Child Health and Human Development, UNITED STATES

## Abstract

Diamond Blackfan Anemia (DBA) is a rare, congenital erythrocyte aplasia that is usually caused by haploinsufficiency of ribosomal proteins due to diverse mutations in one of several ribosomal genes. A striking feature of this disease is that a range of different mutations in ribosomal proteins results in similar disease phenotypes primarily characterized by erythrocyte abnormalities and macrocytic anemia, while most other cell types in the body are minimally affected. Previously, we analyzed the erythrocyte membrane proteomes of several DBA patients and identified several proteins that are not typically associated with this cell type and that suggested inflammatory mechanisms contribute to the pathogenesis of DBA. In this study, we evaluated the erythrocyte cytosolic proteome of DBA patients through in-depth analysis of hemoglobin-depleted erythrocyte cytosols. Simple, reproducible, hemoglobin depletion using nickel columns enabled in-depth analysis of over 1000 cytosolic erythrocyte proteins with only moderate total analysis time per proteome. Label-free quantitation and statistical analysis identified 29 proteins with significantly altered abundance levels in DBA patients compared to matched healthy control donors. Proteins that were significantly increased in DBA erythrocyte cytoplasms included three proteasome subunit beta proteins that make up the immunoproteasome and proteins induced by interferon-γ such as n-myc interactor and interferon-induced 35 kDa protein [NMI and IFI35 respectively]. Pathway analysis confirmed the presence of an inflammatory signature in erythrocytes of DBA patients and predicted key upstream regulators including mitogen activated kinase 1, interferon-γ, tumor suppressor p53, and tumor necrosis factor. These results show that erythrocytes in DBA patients are intrinsically different from those in healthy controls which may be due to an inflammatory response resulting from the inherent molecular defect of ribosomal protein haploinsufficiency or changes in the bone marrow microenvironment that leads to red cell aplasia in DBA patients.

## Introduction

Diamond Blackfan Anemia (DBA) is a rare, macrocytic anemia that affects approximately seven per million live births and is characterized by a paucity of erythrocyte precursors and congenital abnormalities in approximately 35% of patients [1]. DBA presents in early infancy, with patients traditionally diagnosed within the first year of life. In approximately 50–60% of DBA patients, the disease is known to be due to mutations or deletions in one of several ribosomal genes that cause haploinsufficiency of ribosomal proteins in either the small or large ribosomal subunits. Mutations have been found in *RPS19*, *RPL5*, *RPS10*, *RPL11*, *RPL35A*, *RPS26*, *RPS24*, *RPS17*, *RPS7*, *RPL26*, and *RPL15* [2–3]. Previously reported red cell characteristics include elevated fetal hemoglobin levels and increased erythrocyte adenosine deaminase activity. Recently, an essential erythropoietic transcription factor, *GATA1*, has also been associated with DBA after the discovery of rare X-linked mutations in DBA patients [4] and it has been shown that decreased translation of the *GATA1* mRNA transcript can occur due to ribosomal haploinsufficiency [5]. Despite advances in whole genome sequencing and the detection of large gene deletions, the genetic cause of DBA is still unknown in approximately 35% of patients [6]. The severity of the disease can vary substantially and some of this heterogeneity may be attributable to underlying genetic variability of DBA. However, even within families with the same ribosomal protein mutation, there is often no apparent correlation between the specific gene mutation and phenotypic severity [7]. As a result, treatment of DBA is variable with patients ranging from transfusion-dependent to corticosteroid-responsive, with 15% of patients undergoing complete hematological remission due to an unknown mechanism [8–9].

Despite a fairly well-defined genetic characterization of DBA, the mechanism by which ribosome haploinsufficiency leads to erythroid-specific defects is still not clear. We hypothesize that protein translation defects may be altered in DBA, leading to abnormalities in erythropoiesis that can affect protein sorting and degradation during erythrocyte maturation. Therefore, investigation into the erythrocyte proteome, to identify altered protein levels in mature red blood cells of DBA patients, may provide insights that are not possible with gene expression analysis. Previously, we had analyzed the erythrocyte membrane proteome of patients with DBA and uncovered several proteins that were present in DBA patients but not healthy donors [10]. The most consistent difference was the presence of dysferlin, a membrane repair protein, in the erythrocyte membrane of DBA patients but not healthy donors. Other proteins in the membranes of DBA patients included several molecules from the major histocompatibility complex (MHC) class I pathway, indicating the involvement of the immune system in the pathogenesis of DBA. We now extend this analysis to the erythrocyte cytosolic proteome to obtain a complete picture of the altered protein composition in the erythrocytes of DBA patients.

Analysis of the erythrocyte cytosolic proteome is complicated by the presence of a large amount of hemoglobin, which makes up over 95% of the cytoplasmic protein content. Despite the large quantity of hemoglobin in the erythrocyte, hundreds of other cytosolic proteins are present and critical for maintaining erythrocyte function and homeostasis. In order to effectively investigate the cytoplasmic erythrocyte proteome, it is essential to decrease the hemoglobin content to enable the detection of lower abundance cytoplasmic proteins. Previous studies have used pre-fractionation of the erythrocyte cytoplasm to separate hemoglobin by employing 1D SDS-PAGE [11], size exclusion chromatography [12], cation exchange chromatography [13], in-solution isoelectric focusing [14], and affinity chromatography [15]. These approaches varied in depth of analysis and enabled detection of up to 700 unique erythrocyte cytoplasmic proteins, however they have drawbacks such as limited loading capacity and loss of proteins co-fractionating with hemoglobin. An elegant alternative approach used peptide ligand library technology to reduce the concentration of hemoglobin, enabling the detection of over 1500 proteins in a series of LC-MS/MS experiments that included LC-MS/MS runs of 20 tryptic digest fractions for three different elution conditions from two hexameric peptide ligand libraries [16]. While this approach led to an impressive number of protein identifications, this study required extensive mass spectrometer time and labor-intensive methods that are difficult to implement for label-free quantitative comparison of multiple small volume blood samples. Alternatively, hemoglobin (Hb) depletion by nickel chelation is a straightforward strategy that improves the detection of lower abundance erythrocyte cytosolic proteins. A study by Ringrose et al described the detection of 700 unique erythrocyte cytosolic proteins after concurrent depletion of hemoglobin with Ni-NTA super-flow columns and carbonic anhydrase 1 with affinity chromatography [15]. To simplify the sample preparation workflow, we focused on Hb-depletion from the erythrocyte cytoplasm using Ni-NTA super-flow columns and found that in-depth analysis could be achieved without the need for either carbonic anhydrase depletion or extensive fractionation.

In this study, we first validated the utility of Ni-NTA columns to effectively and reproducibly deplete hemoglobin in a manner that is amenable to an in-depth analysis of the erythrocyte cytosol proteome using small volume clinical blood samples. Effects of varying degrees of fractionation and LC-MS/MS analysis parameters on depth of analysis of the Hb-depleted cytosolic erythrocyte proteome, as well as reproducibility of Hb-depletion using nickel columns were assessed. An optimized method was developed that could reliably quantitate over 1000 proteins from Hb-depleted erythrocyte cytosols. This method was then used to investigate changes in the erythrocyte cytoplasmic proteome of patients with DBA compared to healthy donors using label-free quantitation to identify a unique inflammatory signature in DBA erythrocytes.

## Materials and Methods

### Erythrocyte Sample Preparation

All experiments were in accordance with procedures approved by the IRB of the Children’s Hospital of Philadelphia and the University of Pennsylvania. Approximately 3–8 mL of peripheral blood in EDTA tubes were obtained from patients and healthy individuals with informed written consent according to the Declaration of Helsinki. Informed written consent from the next of kin, caretakers, or guardians was obtained on behalf of the minors enrolled in this study. Erythrocytes were purified as previously described using leukocyte depletion and buffy coat removal to minimize protein contributions from contaminating cell types [17]. After washing the cells with PBS they were lysed with 10 volumes of hypotonic lysis buffer and membranes were separated with centrifugation at 31,000xg for 30 minutes. The supernatant containing the cytoplasm was snap frozen and stored in aliquots at -80°C until use.

### Hemoglobin Depletion with Ni-NTA Columns

Depletion of hemoglobin from erythrocyte cytosol was modified from a previously published study by Ringrose et al [15]. Ni-NTA Superflow Columns (Qiagen, Venlo, Netherlands) were equilibrated with 10 ml NPI-10 buffer (50 mM NaH_2_PO_4_, 300 mM NaCl, 10 mM imidazole, pH 8.0). 2 mL erythrocyte cytoplasm was loaded onto the Ni-NTA columns with gravity flow. Cytosolic proteins were eluted six times with 1 mL NPI-15 buffer (50 mM NaH_2_PO_4_, 300 mM NaCl, 15 mM imidazole, pH 8.0). In cases where detectable hemoglobin eluted during the NPI-15 washes, the red eluate was subjected to one more round of Hb-depletion in new equilibrated Ni-NTA columns and washed two times with 1 mL NPI-15. The Hb-enriched fraction was collected with four elutes of 1 mL NPI-250 buffer (50 mM NaH_2_PO_4_, 300 mM NaCl, 250 mM imidazole, pH 8.0). The fraction containing the Hb-depleted cytosolic proteins was concentrated to approximately 2 mg/mL using 3 kDa molecular weight cutoff protein concentrators (Thermo Scientific, Rockford, IL). The final protein concentration was determined using a Modified Lowry Protein Assay kit (Thermo Scientific). Samples were analyzed by SDS-PAGE on 12-well 10% Bis-Tris gels (Invitrogen, Carlsbad, CA) followed by staining with colloidal Coomassie (Invitrogen).

### Sample Preparation for LC-MS/MS

For preliminary analyses of the total erythrocyte cytoplasm as well as the Hb-depleted and Hb-enriched fractions of cytoplasm, 13 μg of protein was run to 1 cm on a 10-well 10% Bis-Tris SDS PAGE gel with MOPS buffer (Invitrogen). For further optimization of Hb-depleted fractions and comparison of Hb-depleted erythrocyte cytoplasm from DBA patients and healthy donors, 25 and 30 μg of protein, respectively, was run to 1 cm on a 10% Bis-Tris SDS PAGE gel with MES buffer (S1 Fig). In-gel trypsin digestion was performed as previously described [17]. Briefly, gel lanes were cut into 12, 1-mm slices (to account for gel swelling) and three adjacent slices from the same sample lane were pooled for in-gel protein reduction and alkylation with tris(2-carboxyethyl)phosphine and iodoacetimide, respectively, followed by trypsin digestion with 0.8 μg sequence grade modified trypsin (Promega, Madison, WI) for 16 hours at 37°C. Final protein digests were pooled for analysis of one or multiple fractions and stored at -20°C prior to LC-MS/MS analysis.

### LC-MS/MS Data Acquisition

LC-MS/MS analysis was carried out using a nanoAcquity HPLC (Waters, Milford, MA) and a LTQ-Orbitrap XL mass spectrometer (Thermo Scientific). For each analysis, 5 μL of tryptic digest was loaded onto a 180 μm x 20 mm trap column packed with 5 μm Symmetry C18 resin (Waters) using solvent A (0.1% formic acid in Milli-Q H_2_O [Millipore, Billerica, MA]) followed by separation on a 75 μm x 250 mm analytical column packed with 1.7 μm BEH130 C18 resin (Waters). Peptides were eluted with either a 4 or 8 hour chromatographic gradient using solvent A and solvent B (0.1% formic acid in acetonitrile). For the 4 hour gradient, peptides were eluted using 5–28% B over 170 minutes, 28–50% B over 50 minutes, 50–80% B over 10 minutes, constant 80% B for 10 minutes, and 80–5% B over 5 minutes. For the 8 hour gradient, peptides were eluted using 5–28% B over 350 minutes, 28–50% B over 150 minutes, 50–80% B over 20 minutes, constant 80% B for 10 minutes, and 80–5% B over 5 minutes. A 30 minute blank gradient was run in between each sample injection to minimize carryover. Full scans were carried out from 400–2000 *m/z* with 60,000 resolution. MS2 data were acquired in data-dependent mode of the top six most intense ions with dynamic exclusion enabled for 60 s, monoisotopic precursor selection enabled, and single charged ions rejected.

### Label-free Quantitation with MaxQuant

Raw data was processed through the MaxQuant Software package (Ver. 1.4.0.8 and Ver. 1.5.2.8) [18]. Proteins were identified with the Andromeda search engine [19] within the MaxQuant program using the human UniRef 100 database (Ver. March 2013) that was expanded to include common environmental contaminants. The search was performed with full tryptic specificity, two missed cleavages, and a fragment ion mass tolerance of 0.5 Da and parent ion tolerance of 20 ppm. Carbamidomethyl cysteine was set as a fixed modification and methionine oxidation and N-terminus acetylation were set as variable modifications. The protein and peptide false discovery rates were both set to 1%, as determined by the Andromeda software using a reverse database, with actual initial false discovery rates of 0.96% and 0.93% for the Hb-depletion preliminary dataset and DBA study, respectively. For all analyses, low confidence proteins that were only identified by one peptide were filtered out yielding final false discovery rates in the range of 0.12 to 0.93%. For preliminary analysis of the utility and reproducibility of Hb-depletion as well as triplicate analysis of Hb-depleted cytosol, label-free quantitation was enabled with no matching between runs. For the large-scale experiment of DBA patients and controls, label-free quantitation was performed with match between runs enabled with a 1 minute matching time window and a 20 minute alignment time window to align features between all samples and allow for quantitation of matching peaks in which no MS/MS was acquired [20]. Initial data filtering and analysis was done using Perseus software to remove reverse hits, proteins identified only by modified peptides, contaminants (trypsin, keratins, etc) and low confidence proteins identified by only one peptide. A full list of peptide and protein identifications as well as label-free quantification measurements of our largest DBA patient dataset can be found in S1 and S2 Tables, respectively. The mass spectrometry proteomics data have been deposited to the ProteomeXchange Consortium [21] via the PRIDE partner repository with the dataset identifier PXD002339.

### Experimental Design and Statistical Rationale

For preliminary analysis of the total erythrocyte cytosol, Hb-depleted fraction, and Hb-enriched fraction, a single blood sample was analyzed to assess the proteome coverage of the total cytosol and depleted fractions. Reproducibility of the Hb-depletion method was assessed using three process replicates of the same blood draw from an individual donor. For comparison of DBA patients and healthy controls, two blood draws from five DBA patients were analyzed along with two process replicates from the same blood draw of one patient (D5) due to sample availability. Six matched healthy donors were chosen for comparison to DBA patients as shown in Table 1. Perseus software was used to impute missing values from normal distribution for statistical analysis. P-values were calculated using a two-tailed student t-test of DBA patients D1-D5 versus all healthy controls, as is appropriate for data with normal distribution. Significantly changed proteins were defined as having both > 3-fold change and a p-value < 0.01. The entire protein dataset was submitted to Ingenuity Pathway Analysis using the Ingenuity Knowledge Base (genes only) as the reference set with direct and indirect relationships included. Filters included human species, experimentally observed or high predicted confidence, and the data sources as follows: BIND, BIOGRID, Breast Cancer Information Core (BIC), Catalogue Of Somatic Mutations In Cancer (COSMIC),Chemical Carcinogenesis Research Information System (CCRIS), ClinicalTrials.gov, Cognia, DIP, Gene Ontology (GO), GVKBiosciences, Ingenuity Expert Findings, Ingenuity ExpertAssist Findings, INTAC, Interactome studies, MINT, MIPS, miRBase, or Obesity Gene Map Database. Significance threshold were set at p < 0.05 and fold change greater than ± 2 and we focused on regulators that had p-values < 0.05 or |Z-score|>1.8 in at least three patients.

**Table 1:** DBA patient and healthy donor demographics.

	Age	Sex	Ethnicity	Gene Mutation	Treatment	Hb* (g/dL)	Erythrocytes*(MIL/μL)	MCV* (fL)
D1	32	F	Caucasian	RPS19, nonsense	None†	4.7, 10.4	1.1, 2.5	116, 119
D2	22	F	Caucasian	RPL5, splice site	None†	10.2, 10.6	3.1, 3.3	93, 93
D3	17	M	Caucasian	Unknown	Prednisone	8.6, 7.0	2.3, 1.9	115, 117
D4	16	M	Caucasian	RPS19, splice site	Prednisone	13.5, 14.3	4.2, 4.3	93, 96
D5	14	M	Caucasian	RPL11, frame shift	Prednisone	11.5	3.7	98
D6	22	F	Caucasian	RPS17, frame shift	Prednisone	n.d, 14.6	n.d, 4.2	n.d, 98
C1	24	F	Caucasian					
C2	28	F	Caucasian					
C3	33	F	Caucasian					
C4	21	M	Caucasian					
C5	22	M	Caucasian					
C6	20	M	Caucasian					

*Two values were from separate blood draws at two different times that were used for the replicate proteome analyses designated as a and b, respectively

n.d—not determined.

Normal values for: Hb—13.5–17.5 males, 12.0–15.5 females; Erythrocytes—4.3–5.7 males, 3.9–5.0 females; mean corpuscular volume (MCV)—80–100 for male and female.

†Patients D1 and D2 were transfused in the past.

### Validation Studies

A number of significantly changed proteins were selected to validate the LC-MS/MS data using immunoblotting. The following antibodies were purchased from Abcam Inc. (Cambridge, MA): anti-TRK fused gene (ab86606), anti-GBP1 (ab119236), anti-N-myc interactor (ab170870), anti-GARS (ab42905), anti-Actn4 (ab108198) and goat anti-mouse HRP conjugate (ab6789). The anti-vinculin antibody (SAB4200080) was purchased from Sigma (St. Louis, MO), an anti-PSMB8 antibody (13635) was purchased from Cell Signaling Technology (Danvers, MA), a goat anti-rabbit HRP conjugate (31460) was purchased from Pierce, and goat anti-mouse Alexa 488 and Goat anti-rabbit Alexa 633 were purchased from Invitrogen for microscopy experiments. To verify consistent sample loading and electrotransfer, the PVDF membranes were stained with Pierce’s MemCode, a reversible protein staining kit for PVDF membranes, (Thermo Scientific) after electrotransfer and before antibody binding, according to the kit instructions. These loading controls for all western blots are shown in S2 Fig. After antibody binding, blots were developed with Pierce ECL-2 Western Blotting substrate (Thermo Scientific). Densitometric quantitation of western blot images was performed using ImageJ software. For immunostaining of erythrocytes, 25 μL of whole blood was washed three times in PBS supplemented with 5 mM glucose. Erythrocytes were fixed in 1 mL of 4% paraformaldehyde in PBS overnight at room temperature. Erythrocytes were then washed three times with PBS supplemented with 0.1 M glycine followed by fixing in PBS/0.1M glycine with 0.2% Triton X-100 for 20 minutes at room temperature. Cells were then washed and resuspended in blocking buffer (PBS, 0.1M glycine, 4% BSA, and 1% goat serum) for at least 2 hours before staining. For staining, 100 μL of resuspended cells were mixed with 100 μL blocking buffer and incubated with 1/100 dilution of primary antibody for 2 hours at room temperature. Cells were washed three times with PBS/0.1M glycine and resuspended in 200 μL blocking buffer and 1/1000 dilution of secondary antibody for a 2 hour incubation followed by 2 washes in PBS prior to imaging. Images were obtained with a Leica TCS Sp5 II scanning laser confocal microscope equipped with AOBS and HyD detectors and a Nikon Eclipse 90i upright fluorescence microscope.

## Results

### Ni-NTA Columns Provide a Reliable and Reproducible Method to Deplete Hemoglobin from Erythrocyte Cytoplasmic Fractions

**Fig 1:**
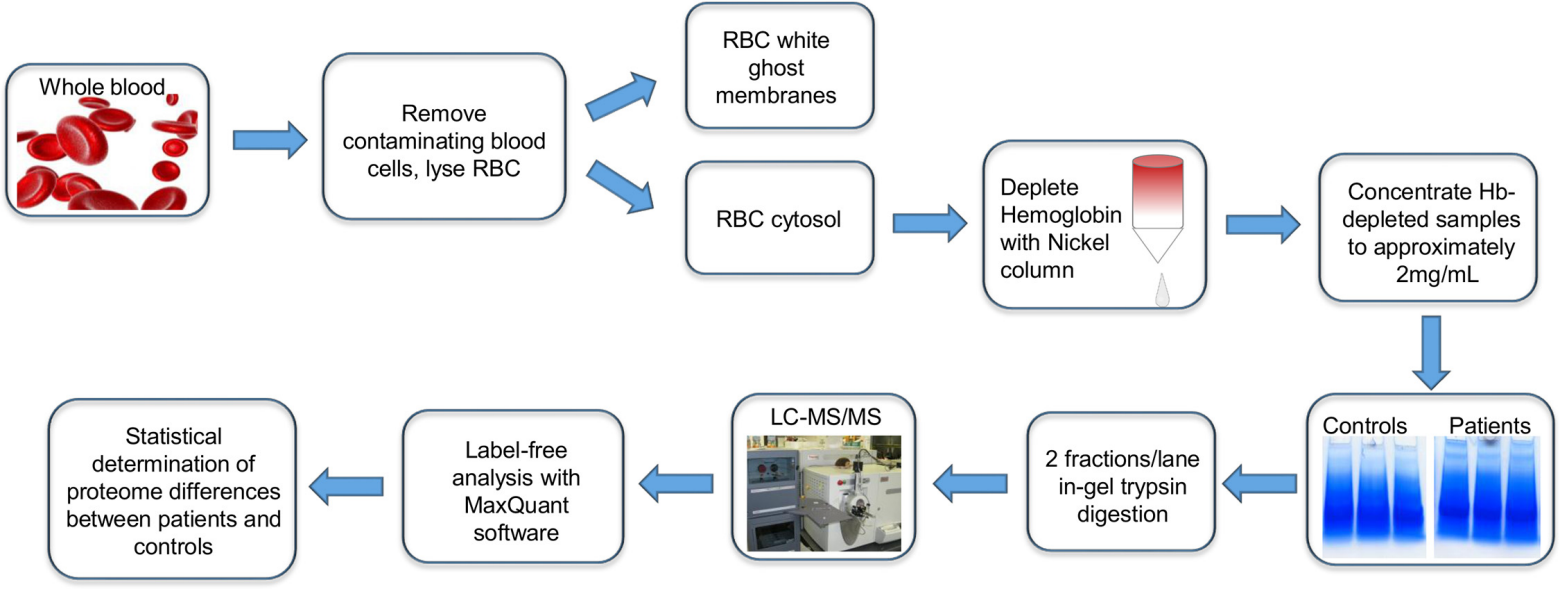
Schematic of erythrocyte purification and cytosolic protein enrichment for label-free proteomic analysis.

As discussed above, proteomics analysis of erythrocyte cytosol is complicated by the very high abundance of hemoglobin. It was therefore necessary to develop an analytical scheme where small volumes of patient blood samples could be analyzed in depth, while simultaneously keeping mass spectrometer analysis time within realistic limits. Therefore, we evaluated the utility of Ni-NTA columns for effective, reproducible depletion of hemoglobin using small blood volumes. We then evaluated the effects of varying degrees of fractionation and LC-MS/MS analysis parameters on depth of analysis of the Hb-depleted cytosolic erythrocyte proteome. Approximately 3–8 mL of peripheral blood in EDTA tubes were obtained from patients and healthy individuals with informed written consent according to the Declaration of Helsinki. Erythrocyte purification was performed as previously described [17] and included leukocyte depletion and elimination of other contaminating cell types by repeated centrifugations and buffy coat removal. Purified erythrocytes were then lysed and separated into membranes (erythrocyte ghosts) and cytoplasm fractions prior to in-gel trypsin digestion and analysis by LC-MS/MS (Fig 1).

**Fig 2:**
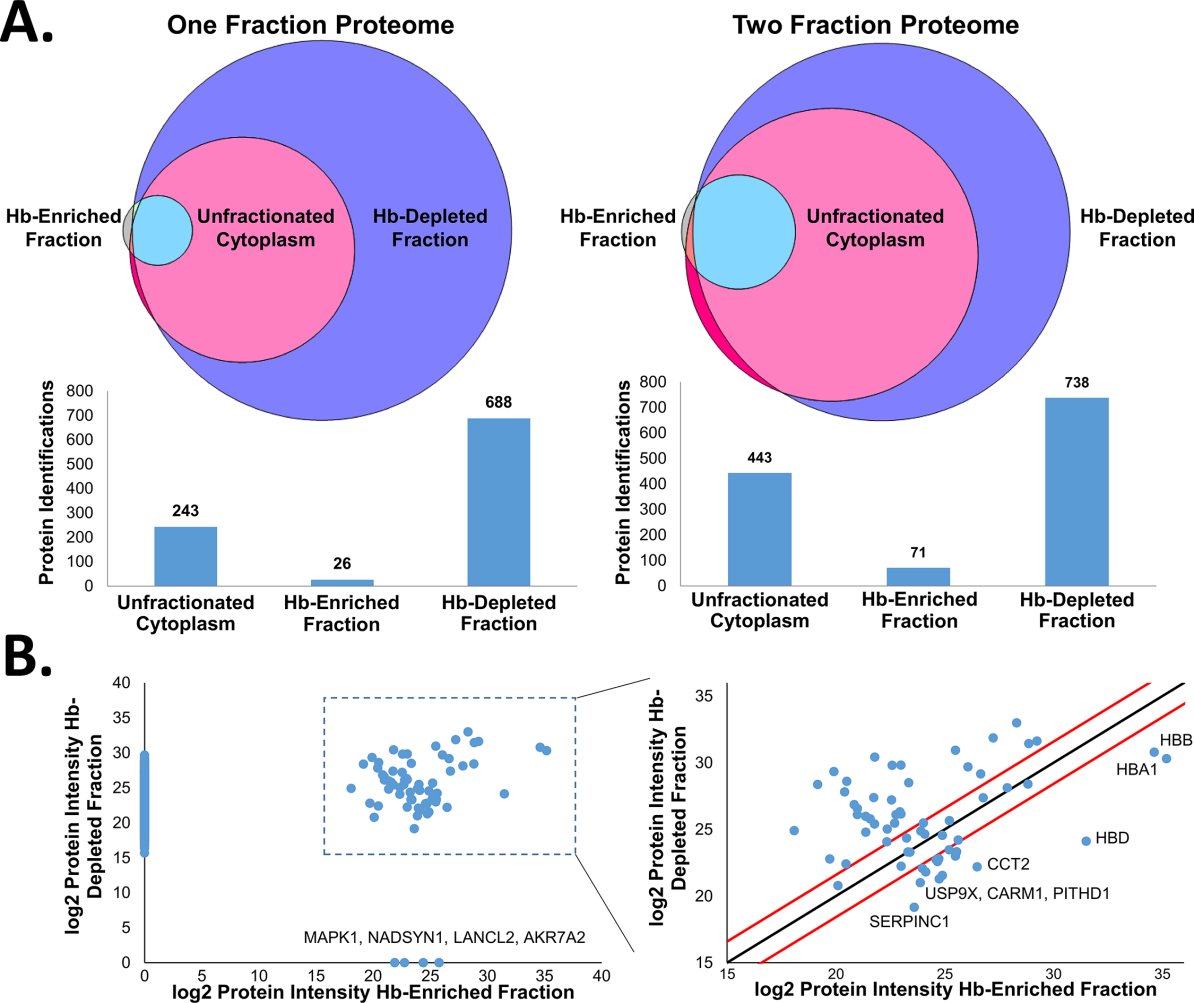
Comparison of protein coverage in Hb-enriched fraction, Hb-depleted fraction, and unfractionated erythrocyte cytoplasm. A) Venn diagram of protein identifications directly proportional to the number of proteins in the overlap using one or two-fraction proteome analyses with corresponding histogram of protein identifications. B) Plots of protein intensity between Hb-enriched and Hb-depleted fractions in the two-fraction proteome, with black line denoting equal protein intensity in each fraction and red lines denoting a three-fold change.

Initial studies were performed to assess the specificity and utility of Hb-depletion using Ni-NTA columns and to evaluate the most efficient trade-off between depth of analysis and mass spectrometer analysis time using blood from a healthy donor. First, the total erythrocyte cytoplasm, Hb-depleted fraction, and hemoglobin eluate were analyzed in either one or two fractions following in-gel trypsin digestion using a 4 hour chromatographic gradient. Results from this analysis showed that as expected, far greater depth of analysis of the cytoplasm could be achieved when hemoglobin was removed, with identification of 688 proteins compared to 243 proteins identified in unfractionated cytosol (Fig 2A). An additional 50 proteins could be identified in the Hb-depleted cytoplasm when the cytosol was separated into two fractions on a short SDS gel. In comparison, only 443 proteins were identified with a two-fraction analysis of the entire cytosol due to the very high abundance of hemoglobin and limited amount of total peptides that can be loaded onto the LC column. The Hb-enriched fraction contained far fewer detectable proteins, with only 26 and 71 proteins identified in the single and two-fraction experiments, respectively (Fig 2B). Importantly, very few non-hemoglobin proteins were completely removed by the Ni-NTA column, as nearly all of the proteins identified in the Hb-enriched fraction were also identified in the Hb-depleted fraction. Comparison of the proteins that were identified in both the Hb-enriched and Hb-depleted fractions indicated that most proteins had a higher intensity in the Hb-depleted fraction, showing they were not enriched in the Hb-enriched fraction, suggesting only incidental retention on the column in a non-specific manner (Fig 2B). Furthermore, the three highest intensity proteins in the Hb-enriched fraction are all hemoglobin proteins, as expected. There are only four proteins that were found in the Hb-enriched fraction and were not detected in the Hb-depleted fraction (on X-axis in left panel of Fig 2B), as well as a handful of proteins that were higher intensity in the Hb-enriched fraction. This minor association of non-Hb proteins with the Ni-NTA column was somewhat variable. In fact, across four different Hb-depletion experiments, there were no proteins that were consistently found in the Hb-enriched fraction that were not detected in the Hb-depleted fractions. These results indicate that the depletion strategy is fairly specific for binding Hb and allows for improved depth of analysis of the erythrocyte cytoplasmic proteome.

**Fig 3:**
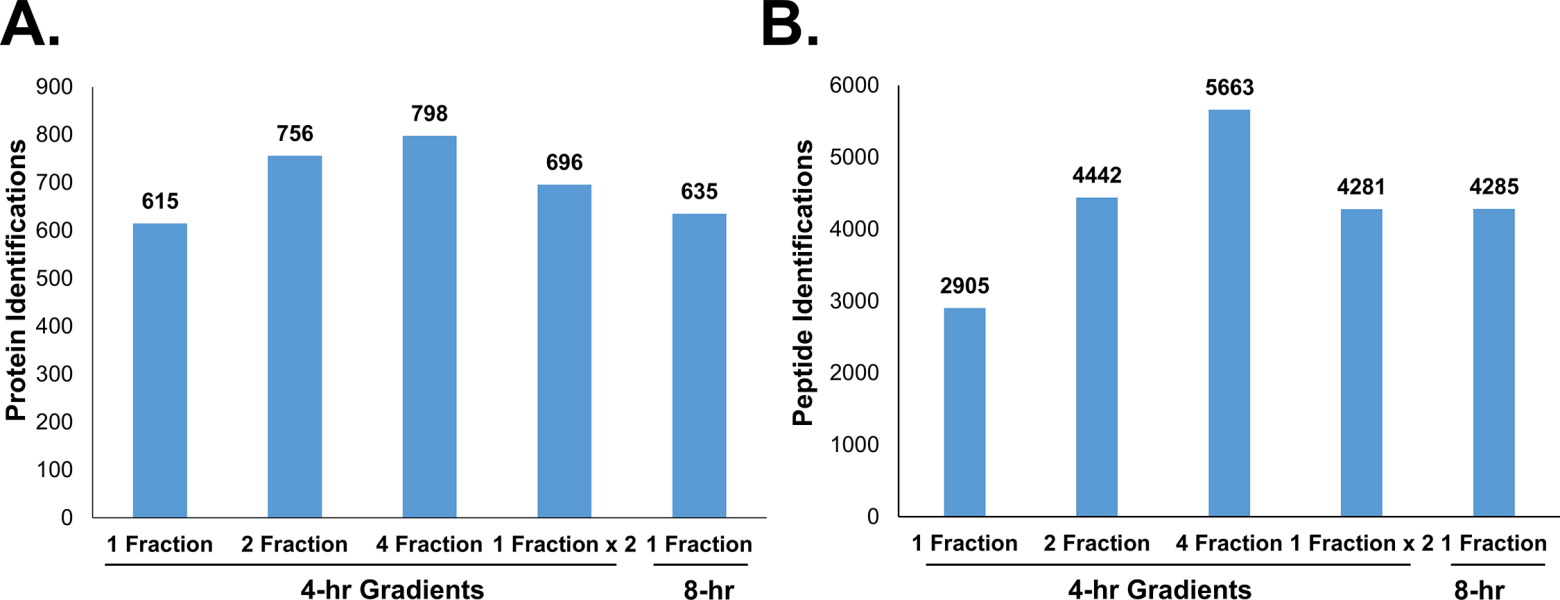
Effects of gradient length and extent of proteome fractions on protein and peptide identifications using Hb-depleted samples. Replicate proteomes were analyzed using a 4-hour gradient to analyze an unfractionated sample or a sample fractionated into two or four fractions. The unfractionated sample also was analyzed using duplicate 4-hour gradients or a single 8-hour gradient. A) Protein identifications across Hb-depleted datasets. B) Peptide identifications across Hb-depleted datasets.

Next we ran a series of experiments to determine the optimal trade-off between total instrument time per sample and depth of analysis when analyzing the Hb-depleted sample using blood from a healthy donor. Length of gradient and number of fractions per proteome were evaluated. In this experiment, analysis of a single fraction proteome using a 4 hour LC-MS/MS run provided 615 proteins. However, a two-fraction proteome was substantially superior with 756 proteins identified, while a four-fraction analysis identified 798 proteins (Fig 3A). Duplicate analysis of a single fraction proteome used the same amount of mass spectrometer time as analysis of a two fraction proteome but less depth of analysis than the two fraction proteome, with only 696 proteins identified. We also tested a single-fraction proteome using an 8-hour chromatographic gradient, which did not improve performance with only 635 proteins identified. While the most peptides were identified in the four-fraction analysis (Fig 3B), this increased depth of analysis only contributed to 42 more protein identifications, which was regarded as insufficient to justify the doubled mass spectrometer time. Therefore, based on these experiments, the best trade-off in depth of analysis and instrument time was regarded as analysis of Hb-depleted cytosol separated into two-fractions and analyzed using 4-hour LC-MS/MS runs.

To ensure that in-depth analyses of patient erythrocyte cytosols would not be biased by contaminating cell types, we searched our results for known abundant proteins from other blood cell types. First, we could not detect any of the blood cell-type specific proteins reported by Haudek et al [22]. We then looked for other known abundant proteins from plasma, platelets, and reticulocytes (albumin, immunoglobulins, fibrinogen, transferrin, etc) and were only able to detect serum albumin with two peptides. These results confirm that the cell population used for proteome analysis was highly enriched for erythrocytes and that contaminating cell types did not significantly contribute to the proteomes identified in this study.

**Fig 4:**
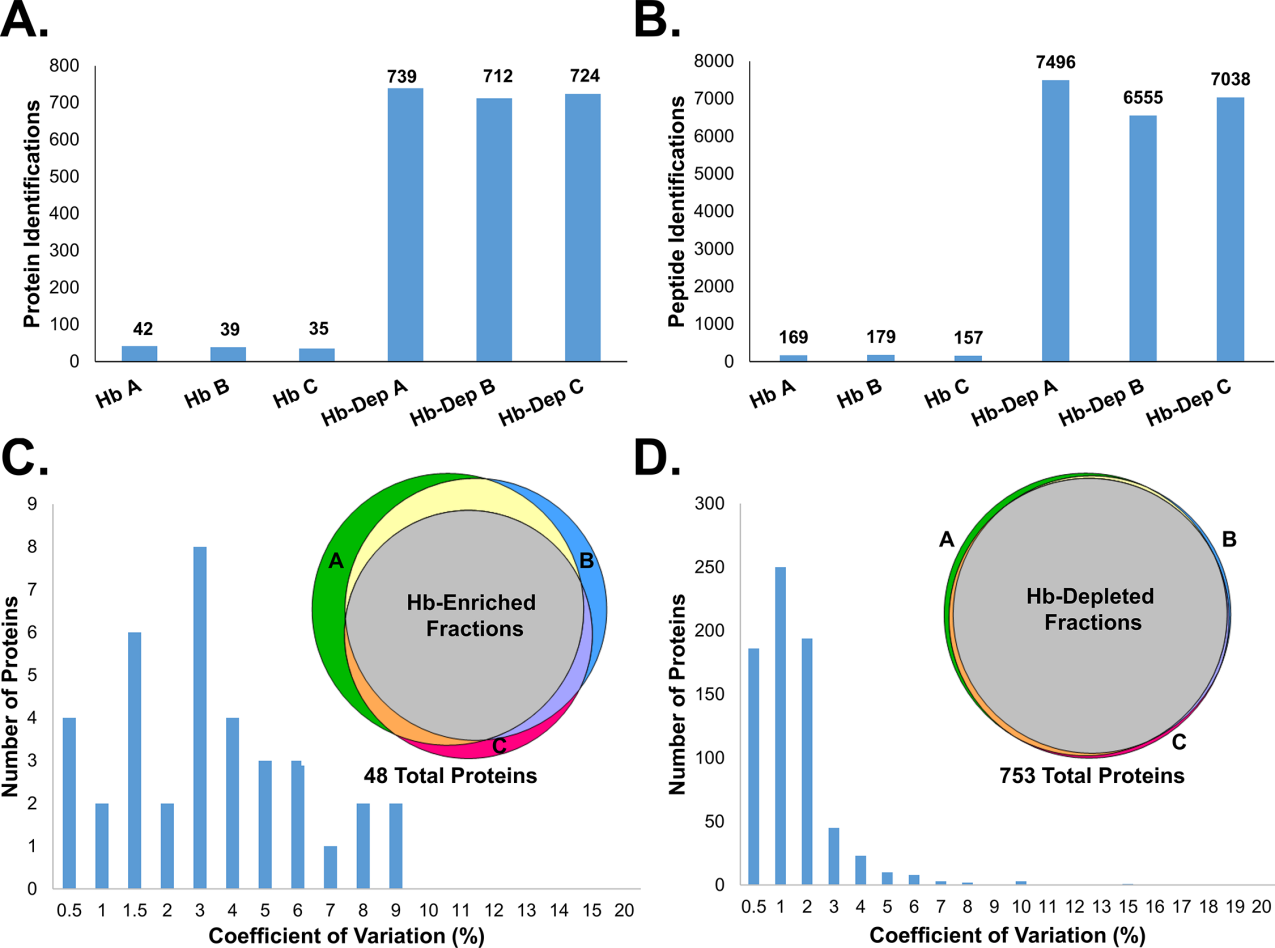
Triplicate Hb-depletions show high reproducibility. A) Number of protein identifications in Hb-enriched fractions and Hb-depleted fractions from triplicate depletions of a single erythrocyte cytosol preparation. B) Number of peptide identifications in these triplicate Hb depletions. C) Histogram showing coefficient of variation in protein intensity across triplicate analyses of Hb-enriched fractions with embedded Venn diagram showing protein overlap between these three datasets. D) Histogram of the number of proteins with corresponding coefficient of variation for triplicate Hb-depleted fractions with Venn diagram showing almost complete overlap of over 750 proteins.

To test the reproducibility of Hb-depletion with Ni-NTA columns, triplicate Hb-depletion was performed using three aliquots of the same erythrocyte cytosol preparation and three Ni-NTA depletion columns. Each Hb-depleted fraction and Hb-eluate was separated into two fractions on short SDS gels and resulting tryptic digests were analyzed by LC-MS/MS. Results from this experiment confirmed that Hb-depletion with Ni-NTA columns is quite reproducible with similar protein and peptide identifications across the triplicate datasets (Fig 4A and 4B). The reproducibility of protein intensities across triplicates was also excellent with coefficients of variation below 5% for most proteins in both the Hb-enriched fractions and Hb-depleted fractions (Fig 4C and 4D). The overlap of protein identifications in triplicate analysis of the Hb-enriched fractions and Hb-depleted fractions also was highly reproducible, especially for the Hb-depleted fractions that have almost complete overlap in protein identifications (Fig 4C and 4D). The larger variation of protein identifications in the Hb-enriched fractions is due to increased variability in detecting lower abundant proteins in the presence of a few very high abundance proteins. Specifically, 88% of the MS/MS spectra that resulted in peptide identifications in the Hb-enriched fraction were for hemoglobin proteins. These results indicate that Hb-enriched depletion with Ni-NTA columns is a very reproducible and reliable way to increase protein identifications of erythrocyte cytoplasmic proteins in a manner that is highly amenable to label-free quantitation.

### Analysis of the Erythrocyte Cytoplasmic Proteome in Diamond-Blackfan Anemia

We used the analytical pipeline described above to assess changes in the erythrocyte cytoplasmic proteome that occur in patients with DBA. Erythrocyte cytosol from six transfusion-independent DBA patients and six healthy donors were each depleted of hemoglobin, concentrated to approximately 2 mg/mL, fractionated, digested with trypsin, and analyzed by LC-MS/MS. Healthy donors were matched to the patients’ demographics based on race, age, and gender (Table 1). Blood drawn on two different dates was analyzed for all DBA patients with the exception of patient D5 where duplicate aliquots from the same blood draw were used because only one blood draw was available. A 30 μg aliquot of protein from each Hb-depleted erythrocyte cytosol was separated into two fractions on short SDS gels and subjected to in-gel trypsin digestion and LC-MS/MS.

**Fig 5:**
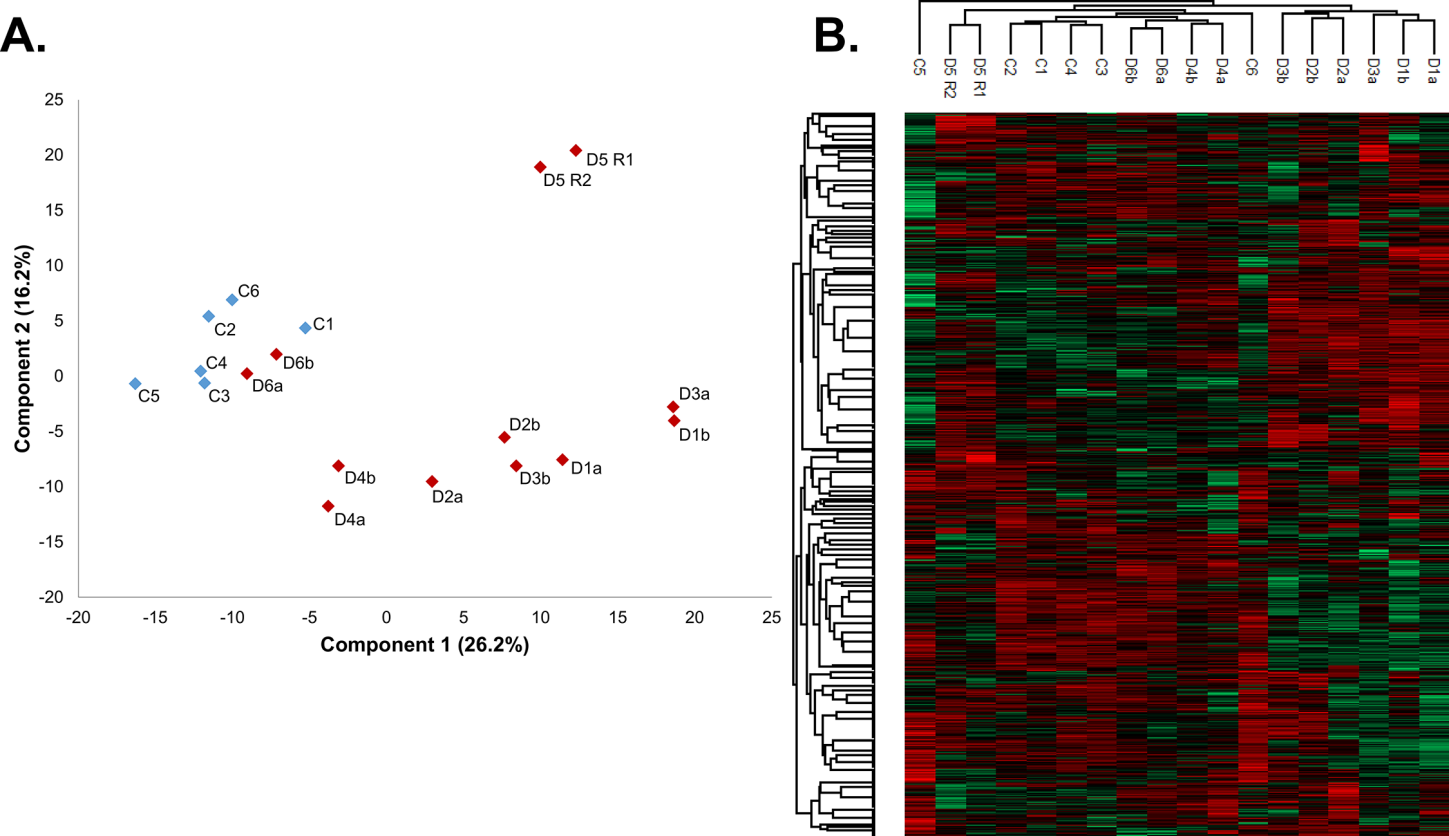
Principal component analysis and hierarchal clustering of DBA and healthy control samples. A) Principal component analysis shows a clear distinction between DBA and control cytosols with the exception of patient D6 which has a protein profile similar to that of healthy donors. B) Hierarchal clustering shows separation between most patients and controls.

Label-free quantitation and data normalization was performed using the MaxQuant software package with matching between runs to yield a final list of 1052 proteins (S2 Table). Principal component analysis and hierarchal clustering, using the intensities of all 1052 proteins, showed good separation between most DBA patients and controls, indicating an inherent difference between the proteomes in each cohort. The most obvious exceptions were the proteomes of patient D6, which clustered with the healthy donor proteomes (Fig 5). This similarity was consistent with the patient’s complete blood counts, which were well within the normal range, indicating that the patient had undergone spontaneous remission (Table 1). Similarly, clinical blood parameters for patient D4 were mostly within the normal range, although Hb values were at the lower boundary, and principal component analysis showed this patient’s proteome was closer to the controls than the remaining DBA patients. These observations suggest that with the recovery to normal blood count values, DBA patients’ erythrocyte cytoplasm proteomes resemble normal proteomes. As patient D6 appeared to be normal based on both clinical parameters and total proteome, data from this patient were not further considered when comparing DBA and control proteomes. Interestingly, all controls were tightly clustered regardless of gender or age, suggesting that all controls could be treated as a single homogeneous group. Not surprisingly, the DBA patient proteomes were more heterogeneous, although the two different blood draws from the same patient were usually closely clustered in the principle component analysis plot and hierarchal cluster analysis (Fig 5). Due to the small variation seen between the male and female control donors in the principal component analysis, all subsequent comparisons were performed between all DBA patients (excluding patient D6) versus all controls.

**Fig 6:**
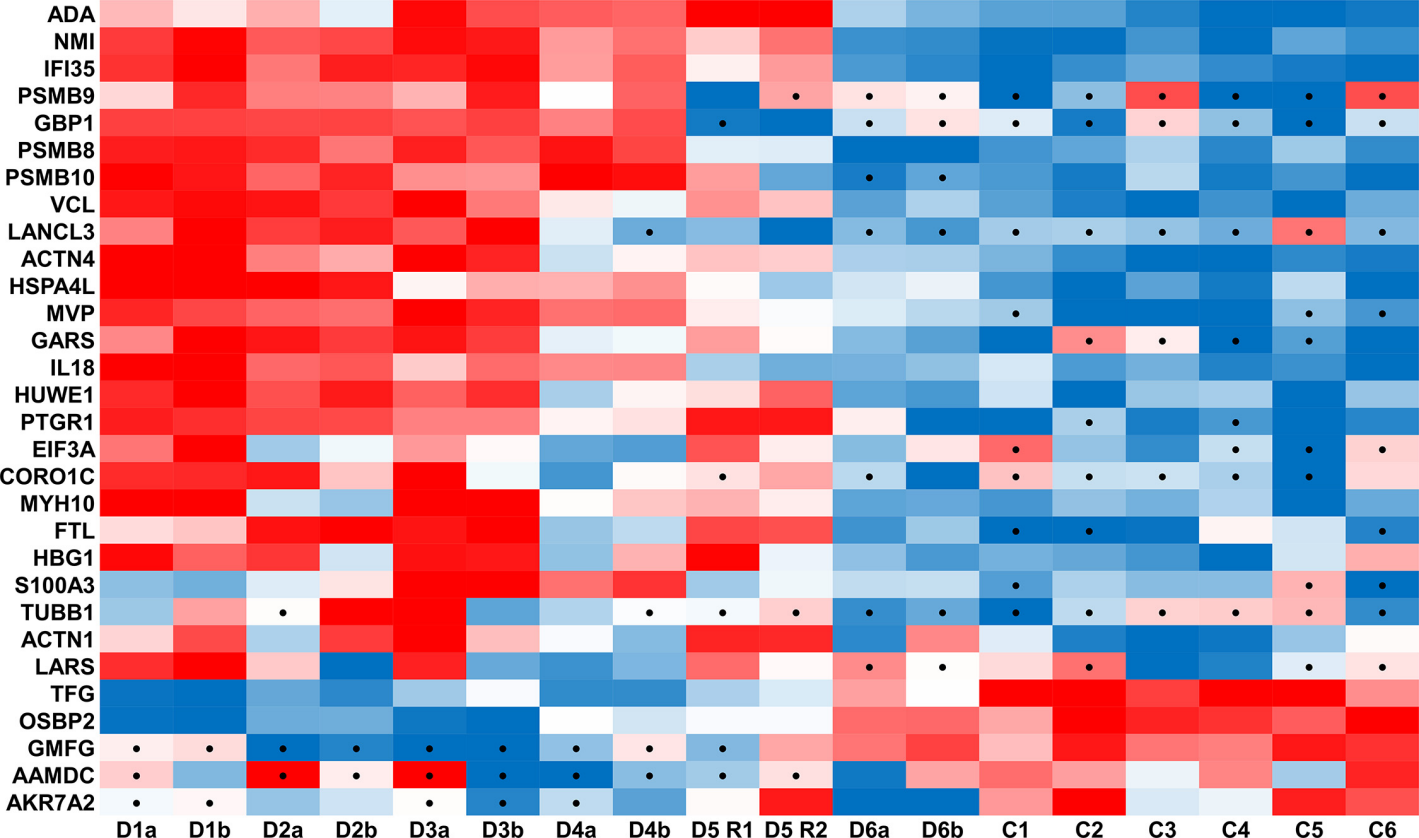
Heatmap of significantly changed proteins. Protein intensity z-scores of significantly changed proteins listed in Table 2. Red denotes high relative protein intensity while blue indicates low intensity with black dots signifying zero values that were imputed for statistical analysis.

**Fig 7:**
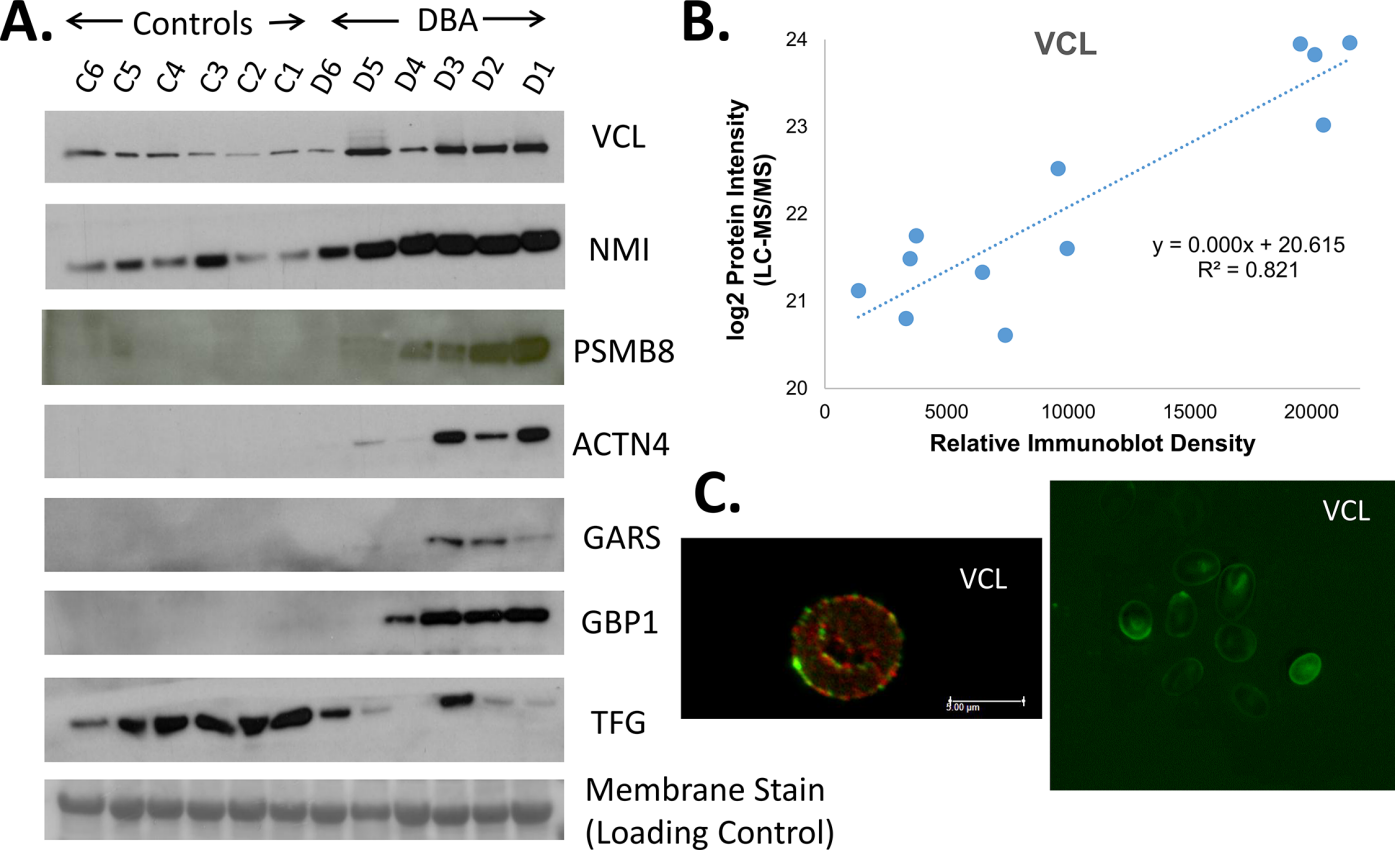
Validation of proteomics data using western blot and immunofluorescence. A) Immunoblot of a number of proteins that were significantly changed in Hb-depleted erythrocyte cytoplasm between DBA patients and healthy donors. Equal protein loading and consistent electrotransfer of samples for the western blots were confirmed by staining the entire PVDF membrane using MemCode, a reversible total protein stain, immediately prior to performing the western blot as shown by the representative 30 kDa protein visualized by membrane stain in the bottom panel. Images for MemCode staining of all other immunoblots are shown in S2B Fig) Correlation between VCL intensity values from LC-MS/MS label-free quantitation and relative density from immunoblot analysis. C) Immunofluorescence of VCL and Band 3 within erythrocytes from patient D3 visualized with confocal microscopy (left, Band 3 in red and VCL in green) and fluorescence microscopy (right, VCL in green).

Statistical analysis was performed to identify proteins that were significantly changed in the DBA patient cohort. Proteins were considered significantly changed if the difference was significant as determined by the student t-test (p < 0.01) and greater than 3-fold (Table 2). Based on these criteria, far more proteins were significantly increased in DBA patients (24 proteins) than showed a decrease (5 proteins) as demonstrated by the heatmap of protein intensity z-scores (Fig 6). Western blots of six proteins that were increased in DBA patients (VCL, NMI, PSMB8, ACTN4, GARS, and GBP1) and one protein that was decreased in DBA patients (TFG) were performed to validate the proteomic label-free data analysis (Fig 7A). Overall, there was good agreement between the western results and label-free quantitation. To further evaluate the consistency between western results and protein quantitation, densitometric values from immunoblots of VCL were compared to LC-MS protein intensities (Fig 7B). A strong positive correlation was observed with an r^2^ of 0.82 (p = 0.001) (Fig 7B). Finally, fluorescence microscopy of cells stained for VCL and erythrocyte membrane protein band 3 confirmed that these proteins could be detected in cells with the expected biconcave shape of erythrocytes (Fig 7C). Similarly, ACTN4 and GBP1 were visualized by immunofluorescence within the cytosol of patient D3, although the signals were weak presumably due to poor antibodies and low protein abundance, respectively (data not shown).

**Table 2:** List of proteins that are significantly increased or decreased in Hb-depleted erythrocyte cytosol of DBA patients.

UniProt Accession Number	Protein names	Gene names	Peptides	p-value	Fold Change
**Known Marker of DBA**					
P00813	Adenosine deaminase	ADA	15	6E-06	3.0
**Increased in DBA Patients**					
Q8WTW2	N-myc-interactor	NMI	5	2E-09	5.3
P80217	Interferon-induced 35 kDa protein	IFI35	6	2E-08	3.5
B0V0T3	Proteasome subunit beta type-9	PSMB9	2	3E-08	37320.8
Q5D1D5	Interferon-induced guanylate-binding protein 1	GBP1	4	1E-07	76290.1
Q6FHU0	Proteasome subunit beta type-8	PSMB8	3	1E-06	3.6
Q6IB22	Proteasome subunit beta type-10	PSMB10	2	2E-06	5.9
K7AE07	Vinculin	VCL	49	6E-06	5.1
Q6ZV70	LanC-like protein 3	LANCL3	4	2E-05	14776.7
O43707	Alpha-actinin-4	ACTN4	46	4E-05	4.2
Q53ZP9	Heat shock 70 kDa protein 4L	HSPA4L	14	9E-05	3.4
Q14764	Major vault protein	MVP	14	0.0001	5397.4
P41250	Glycine-tRNA ligase	GARS	3	0.0002	2963.9
Q14116	Interleukin-18	IL18	7	0.0004	3.6
Q9NXW1	BJ-HCC-24 tumor antigen	HUWE1	4	0.0006	3.1
Q14914	Prostaglandin reductase 1	PTGR1	5	0.0013	629
Q05BS0	Eukaryotic translation initiation factor 3 subunit A	EIF3A	4	0.0017	1308.5
Q9ULV4	Coronin-1C	CORO1C	2	0.0022	3254.7
P35580	Myosin-10	MYH10	65	0.0025	3.8
P02792	Ferritin light chain	FTL	4	0.0044	1011
P69891	Hemoglobin subunit gamma-1	HBG1	17	0.0048	7.6
P33764	Protein S100-A3	S100A3	3	0.0062	588.9
Q9H4B7	Tubulin beta-1 chain	TUBB1	5	0.0078	1644.8
P12814	Alpha-actinin-1	ACTN1	22	0.0078	9.3
F5H698	Leucine—tRNA ligase, cytoplasmic	LARS	3	0.0082	291.9
**Decreased in DBA Patients**					
Q05BK6	Tyrosine-protein kinase receptor	TFG	5	7E-07	-3.7
Q0VF99	Oxysterol-binding protein 2	OSBP2	12	3E-05	-3.7
Q8TDZ6	Glia maturation factor gamma	GMFG	2	4E-05	-28436
Q9H7C9	Mth938 domain-containing protein	AAMDC	2	0.0006	-4913.5
O43488	Aflatoxin B1 aldehyde reductase member 2	AKR7A2	2	0.0056	-1321.7

Ingenuity Pathway Analysis (IPA) software was used to investigate common pathways and upstream regulators. For this analysis, the entire proteomic dataset was submitted to IPA for each patient versus all controls and filtered on p-values less than 0.05 and fold changes greater than two. Comparison of all patient datasets was then performed in IPA to compile the pathways that are significantly altered with p-values < 0.05 or |Z-scores|>1.8 in three or more DBA patients (Table 3). Results from this analysis highlighted upstream regulators, that based on the expression changes of their known downstream genes, are predicted to be inhibited or activated such as mitogen activated kinase 1 (MAPK1) or Interferon-γ, respectively and also tumor suppressor p53 (TP53), a widely accepted regulator in DBA [23–24]. Pathway analysis revealed canonical pathways that were altered in several DBA patients such as activated integrin signaling, altered actin cytoskeletal signaling, and inhibited RhoGDI signaling. In terms of diseases and biological functions, there is a clear involvement of an immune response, altered cell movement and migration, and proliferation of cells. These results give insight into the biological implications of the protein changes that occur in the erythrocyte cytosol of DBA patients and indicate that an inflammatory element is associated with the pathogenesis of DBA that may lead to some of the intrinsic differences in the erythrocyte cytosol proteomes of DBA patients compared to healthy controls.

**Table 3:** Ingenuity Pathway Analysis of Upstream Regulators in Erythrocytes of DBA Patients

	-LOG_10_(p-value)^1^						activation z-score^2^					
Upstream Regulators	D1	D2	D3	D4	D5	D6	D1	D2	D3	D4	D5	D6
MAPK1	2.9	5.0	3.5	4.1	1.5	-	-2.4	-2.6	-2.4	-2.2	-	-
IFNG	0.7	1.3	1.0	-	-	-	1.9	2.0	2.0	-	-	-
EIF2AK2	3.1	5.2	3.5	3.3	1.6	-	2.0	1.4	2.0	-	-	-
TNF	1.4	2.4	1.4	2.2	2.5	-	0.8	0.8	1.2	0.9	0.3	-
TP53	3.4	2.0	2.6	1.3	-	-	-2.2	-0.3	0.7	-	-	-
CEBPA	-	3.7	1.5	1.4	3.8	-	-	-0.4	-	-	2.2	-
MGEA5	3.6	1.7	2.7	3.5	-	-	-	-	-1.6	-0.4	-	-
Lh	7.3	3.4	2.2	-	1.9	-	-2.0	-	-	-	-	-
FSH	6.1	2.8	1.7	-	1.5	-	-2.0	-	-	-	-	-
P38 MAPK	1.9	1.7	1.5	-	-	-	1.2	-	-	-	-	-

^1^ p-values for significance of enrichment of regulator targets among the analyzed gene set.

^2^ Z-scores for prediction of the regulator activity based on their target expression change direction.

## Discussion

In this study we show that Hb-depletion with Ni-NTA columns enables efficient and reproducible in-depth analysis of the cytosolic proteome of erythrocytes using small volumes of blood that are typically readily available during clinical treatment of patients. This depletion strategy is fairly specific for hemoglobin with incidental, partial removal of only a few other proteins. The depth of analysis of the Hb-depleted cytosol could be increased to more than 700 proteins in a single proteome using only moderate instrument analysis time. This depletion strategy is straightforward and should be easily scaled up or automated for larger numbers of samples using devices such as the BioRobot vacuum manifold (Qiagen). Therefore this workflow allows for an in-depth analysis of the erythrocyte cytoplasmic proteome amenable to label-free LC-MS/MS comparisons of clinically-available blood samples.

Our comparison of the erythrocyte cytosolic proteomes of DBA patients and healthy donors using label-free quantitation identified 1,052 total cytosolic proteins. The substantial increase in total proteins identified in the DBA patient/control study compared with preliminary method optimization experiments on individual donor samples is due to the presence of many low abundant proteins near the detection limit that were variably detected in different proteomes. This is illustrated by the fact that only 601 proteins were identified by at least one MS/MS spectra in each of the 18 analyses and 719 proteins were identified in 15 or more analyses of DBA patients and controls. Also, there were 115 proteins that were not identified in any of the controls at the MS/MS level. However, at the label-free signal matching level, only 8 proteins were apparently unique to DBA patients, indicating that most proteins not identified in any control at the MS/MS level were present with signals in MS1 scans. This indicates that there are a large number of proteins in the cytosols that are near the detection limit of the method used. Many of these low abundance proteins appear to be stochastically identified in only a subset of the samples, as is typically the case for detection of low abundance proteins in complex samples when using LC-MS/MS. This apparent stochastic detection of low abundant proteins may be accentuated by donor-to-donor and blood draw-to-blood draw variations of some of these low abundance proteins, causing them to fall below detection limits in some samples. We did show that doubling the analysis to four fractions did moderately increase the number of proteins identified and substantially increased the number of peptides identified (Fig 3). While analysis of larger numbers of fractions probably would reduce the highly variable detection of low abundance proteins, this would greatly increase total instrument time required. Overall, the current method represents a good compromise between depth of analysis and instrument time when analyzing a substantial number of samples and provides sufficient depth of analysis to gain insights into the biology of DBA as discussed below.

Interestingly, the majority of significantly changed proteins were increased in DBA patients. Out of the 29 proteins that were found to be significantly changed in DBA patients, 24 of them were increased in DBA patients. In fact, 11 of those proteins were below the detection limit in three or more healthy donors. These results are consistent with our findings from the DBA erythrocyte membrane proteome in which several proteins were found in the erythrocyte membranes of patients with DBA that were not detected in healthy controls [10]. Significantly changed proteins from our study on the erythrocyte membrane were distinct from the cytoplasmic proteins in the current study with the exception of the major vault protein, PSMB8, PSMB9, and hemoglobin gamma that were increased in both the erythrocyte cytosols (Table 2) and membranes of DBA patients [10].

One striking result of this study is the close correlation between patient’s cytoplasmic proteomes and their hematological status. For example, patient D6 has completely normal hematological status and principle component analyses of her proteome shows it is closely related to all normal controls and very different from most other DBA patients, as is also apparent in the heat map of protein intensity z-scores in Fig 6. This is strongly indicative of patient D6 achieving a spontaneous remission and no longer needing treatment despite the persistence of the germline RPS17 frame shift mutation in this patient. Furthermore, patient D4 had borderline normal complete blood count values and an intermediate protein profile between DBA patients and controls. It will be interesting to follow up on these patients to verify that they are entering a state of spontaneous hematological remission. These results further suggest that global proteome level analysis of erythrocyte cytosols or specific proteins in Table 2 could be complementary biomarkers to current clinical parameters for diagnosis and treatment of DBA patients. It is possible that the observed correlation between patient hematological status and erythrocyte proteome signature relates to response to prednisone treatment. However, this important connection was beyond the scope of this study because pre- and post-treatment blood samples were not available for these patients. It is anticipated that future proteomics experiments using blood collected longitudinally from DBA patients starting prior to and throughout corticosteroid treatment will be able to directly address the effects of this therapy on proteome signatures of erythrocytes.

Traditionally, the clinical diagnosis of DBA includes elevated adenosine deaminase (ADA) activity levels in erythrocytes of DBA patients. In our analysis, the protein levels of ADA correlate with DBA, with a significant p-value of 5.5x10^-6^ and a fold change of 2.98. However, the first six proteins lists under ADA in the heat map in Fig 6 (NMI, IFI35, PSMB9, GBP1, PSMB8, PSMB10) have greater fold changes that range from 3.5- to >76,000-fold increases in DBA patients relative to healthy donors. These changes also are more significant with p-values ranging from 2.3x10^-6^ to 2.3x10^-9^. Interestingly, these six proteins have all been shown to be inducible by interferon signaling. NMI and IFI35 are partially homologous proteins that can form a 300–400 kDa cytoplasmic complex upon interferon stimulation [25–26]. GBP1, PSMB8, PSMB9, and PSMB10 have been found to be induced by interferon-γ as secondary-responsive IFNγ-inducible genes [27]. PSMB8, PSMB9, and PSMB10 are all components of the immunoproteasome that replace the constitutive catalytic proteasome subunits β1, β2, and β5 upon treatment with interferon-γ [28]. The change in catalytic proteasomal subunits in the immunoproteasome results in conformational changes of substrate binding pockets[29] to alter catalytic cleavage and subsequently improve the quality and quantity of peptides loaded onto MHC class I molecules [30–31]. The significant increase of these proteasomal proteins in the erythrocyte cytoplasm of DBA patients is consistent with our prior analysis of DBA erythrocyte membrane proteomes which showed MHC class I proteins were significantly increased in DBA patients including HLA-A, HLA-B, B2M, TAP1, TAP2, and TAPBP [10]. The presence of the nearly complete MHC Class I machinery in the erythrocytes of DBA patients suggest that this pathway may be functional in the mature red blood cells but is likely activated in erythrocyte precursors, presumably in response to inflammatory signaling. This is consistent with several reports that have described the presence of MHC Class I machinery in erythrocytes of patients with autoimmune disorders such as systemic lupus erythematosus [32–33].

Our results are also consistent with a recent study by Bibikova et al., who reported a novel role for inflammation in the pathogenesis of DBA [34]. They showed that the knockdown of RPS19 in human hematopoietic progenitor CD34^+^ cells led to a down regulation of *GATA1* in a p53 dependent manner through a TNF-α/p38 MAPK mediated mechanism. Furthermore, they found that inflammatory cytokines were upregulated in several zebrafish models of DBA and RPS19 deficient hematopoietic primary cells. These results support a model of increased inflammatory signaling in response to defective ribosome biogenesis that leads to the down regulation of *GATA1* to subsequently inhibit erythroid differentiation [34]. In fact, it has been shown in multiple cells lines that proinflammatory cytokines can inhibit erythroid differentiation [35–36]. Our results on the increase of the immunoproteasome and MHC Class I proteins in the erythrocytes of DBA patients, as well as the predicted upstream regulators MAPK1, IFNG, TNF, and p38/MAPK support the involvement of inflammatory signaling in the pathogenesis of DBA. Taken together, these proteins create a bio-signature of DBA erythrocytes, which demonstrate that DBA red blood cells are inherently different from those of normal controls and the extent of differences in the proteome reflect the severity of the disease.

There are at least three alternative reasons why proteomes of DBA erythrocytes could differ from those of healthy donors, including: 1) genes that are not normally expressed in erythroid precursors are expressed and persist in the mature erythrocyte; 2) selected proteins normally present in erythroid precursors are incompletely removed during enucleation and erythroid maturation, or 3) proteins normally expressed in erythroid precursors are expressed at a higher level in DBA patients and persist in the mature cell. Our data is most consistent with the third possibility because the total proteome of healthy controls and DBA patients are qualitatively nearly identical; that is, most cytosol proteins shown to be elevated in DBA patients were also detectable in erythrocytes from matched normal donors. Furthermore, mature adult red cells undergo exocytosis, but do not undergo endocytosis via the classic receptor-mediated mechanism, indicating that the observed increases in protein levels resulted from synthesis in red cell precursors and not by uptake through endocytosis [37].

Previous work by Gazda et al investigated the differences in global gene expression between multipotential, erythroid, and myeloid bone marrow progenitor cells from DBA patients and healthy controls [38]. This work highlighted the upregulation of several apoptotic genes including two proteins in the TNF receptor superfamily, TNFRSF10B and TNFRSF6. While these results do not directly correlate with the protein increases described in this study, they are in good agreement with the predicted upregulation of TNF, as found in the pathway analysis. A recent study by Ge et al showed dysregulation of TGFβ signaling in DBA-derived induced pluripotent stem cells in which activation of the TGFβ pathways led to decreased erythroid differentiation [39]. TGFβ signaling has also been shown to exhibit immunomodulatory effects [40] which may be linked to the inflammatory signature found in DBA erythrocytes.

Interestingly, few proteins are decreased in DBA erythrocytes compared to controls but a substantial number are increased. These increased proteins highlight the induction of the immunoproteasome through the MHC Class I pathway and increased inflammatory signaling compared to healthy donors. These protein changes are probably a result of ribosomal stress during erythropoiesis and are likely to play functional roles in inflammatory signaling in erythroid precursors. The presence of the inflammatory proteasome may bear interesting new implications in the pathogenesis of red cell aplasia in DBA and could provide a potential target for therapy that will selectively affect DBA red blood cells. Indeed, selective inhibitors of the inflammatory proteasome have recently become an exciting new target for drug development for the treatment of cancer and other diseases with aberrant immune function [41]. Our analysis does not distinguish whether the induction of the immunoproteasome is due to an aberrant ribosome biogenesis in DBA erythrocytes or a response to inflammatory signals in the bone marrow environment resulting from the abnormal hematopoiesis. Functional studies to distinguish between the two are ongoing.

## Supporting Information

S1 Fig Preparative gels of Hb-depleted erythrocyte cytosols for in-gel trypsin digestion. Protein loading is consistent across DBA patients and healthy donors.
(TIF)

S2 Fig Loading Controls for Immunoblots. PVDF membranes stained with MemCode prior to western blotting to verify consistent protein loading and electrotransfer. Variability in protein band patterns across membranes is due to different percentage gels.
(TIF)

S1 Table Complete list of identified peptides in Hb-depleted cytosol of DBA patients and healthy controls. (XLSX)

S2 Table Complete list of identified proteins with label-free quantitation (LFQ) in Hb-depleted cytosol of DBA patients and healthy controls. (XLSX)
