# Supplementary figures and images for: In-Depth, Label-Free Analysis of the Erythrocyte Cytoplasmic Proteome in Diamond Blackfan Anemia Identifies a Unique Inflammatory Signature
Source: PLoS One. 2015 Oct 16;10(10):e0140036. doi: 10.1371/journal.pone.0140036 (PMC4608755; doi:10.1371/journal.pone.0140036)

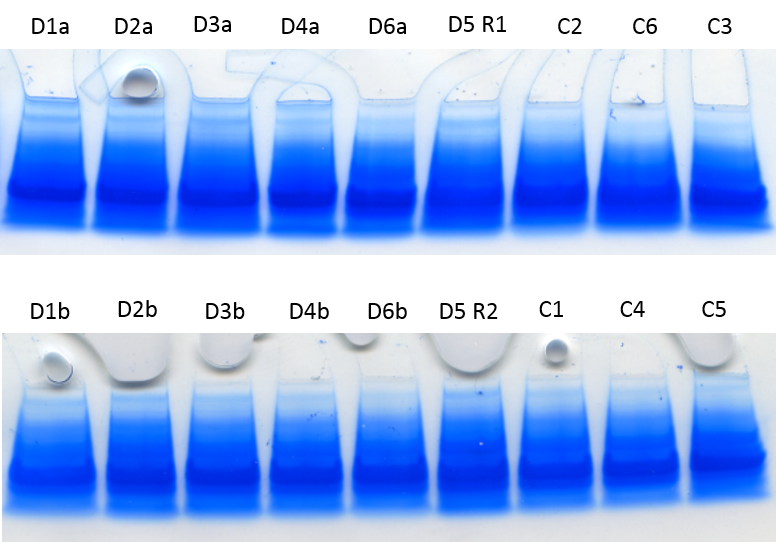

Supplement: S1 Fig — Protein loading is consistent across DBA patients and healthy donors. (TIF) [file pone.0140036.s001.tif]

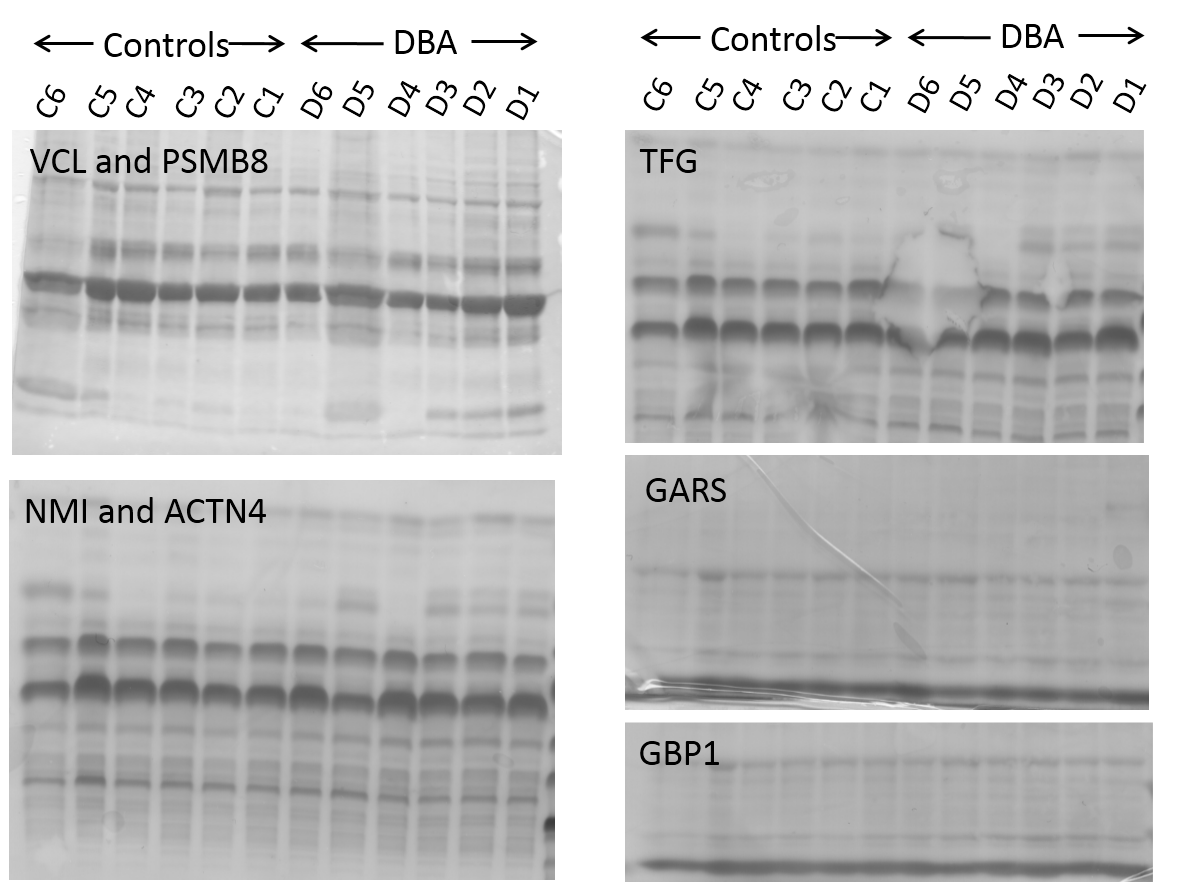

Supplement: S2 Fig — PVDF membranes stained with MemCode prior to western blotting to verify consistent protein loading and electrotransfer. Variability in protein band patterns across membranes is due to different percentage gels. (TIF) [file pone.0140036.s002.tif]
